# Hepatocyte growth factor and myopia: Genetic association analyses in a caucasian population

**Published:** 2009-05-20

**Authors:** Tammy Yanovitch, Yi-Ju Li, Ravikanth Metlapally, Diana Abbott, Khanh-Nhat Tran Viet, Terri L. Young

**Affiliations:** 1 Center for Human Genetics, Duke University Medical Center. Durham, NC; 2 Department of Ophthalmology, Duke University Eye Center. Durham, NC; 3 Department of Biostatistics and Bioinformatics, Duke University Medical Center. Durham, NC

## Abstract

**Introduction:**

Hepatocyte growth factor (*HGF*) and hepatocyte growth factor receptor (*C-MET*) genes have previously been reported to be associated with myopia in Asian family-based and case-control association studies, respectively. We examined whether these genes were associated with myopia in a Caucasian family dataset biased towards high myopia.

**Methods:**

Participating families had at least one offspring with high myopia (≤-5.00 diopters [D]). Genotyping was performed with tagging single nucleotide polymorphisms (SNPs) for each candidate gene using Taqman™ allelic discrimination assays. The data were analyzed with two family-based association methods, the pedigree disequilibrium test (PDT) and the association in the presence of linkage (APL) test. Analyses compared 1) high myopia (<-5.00 D), 2) mild to moderate myopia (-0.50 to -5.00 D), 3) any myopia (<-0.50 D) and 4) extreme high myopia (≤-10.00 D) versus emmetropia using refractive error as either sphere (SPH) or spherical equivalent (SE=sphere + [cylinder/2]). Bonferroni correction was applied to adjust for multiple testing leading to significance levels of 0.0125 for *HGF* and 0.008 for *C-MET*. Two and three-marker sliding window haplotype association tests using APL were also performed for *HGF* markers. Significance levels for haplotype association testing were set at 0.01 for the global tests, and 0.007 for the three marker haplotype specific tests and 0.0125 for the two marker haplotype specific tests.

**Results:**

A total of 146 multiplex families consisting of 649 Caucasian subjects were included. The *HGF* SNP, rs3735520 (APL p=0.002768 for SPH and 0.005609 for SE), and the haplotypes, rs2286194-rs3735520-rs17501108 (APL p=0.007403 for SPH and 0.062685 for SE) and rs12536657-rs2286194 (APL p=0.004219 for SPH and 0.00518 for SE), showed significant association with mild to moderate myopia versus emmetropia. A promising association between extreme high myopia and the *HGF* SNP, rs2286194, was also found (APL p=0.005763 for SPH and 0.004103 for SE). No evidence of association was found in the SNPs tested for *C-MET.*

**Conclusions:**

This study supports a strong association between the mild to moderate myopia group and the *HGF* SNP rs3735520 and the *HGF* haplotypes rs2286194-rs3735520-rs17501108 and rs12536657-rs2286194, and a moderate association of the extreme high myopia with rs2286194. *C-MET* polymorphism statistical associations with myopia in an Asian study were not replicated in our Caucasian cohort. *HGF* may be a potential myopia candidate gene for further investigation.

## Introduction

Myopia affects 33.1% of the American population greater than 12 years of age [1], resulting in dependence on optical correction and/or treatment with refractive surgery. The estimated economic impact of correcting myopic adults in the United States is $3.8-7.2 billion annually [2]. In addition to financial burdens, studies have shown decreased quality of life associated with higher levels of myopia in patients wearing spectacle correction and/or contact lenses [3]. Patients with pathologic myopia also have increased risk of premature cataract, glaucoma, retinal detachment, and chorio-retinal degeneration [4].

The mechanism underlying myopia is thought to be due to molecular changes in the sclera via a process known as “scleral remodeling” [4]. During this process, thinning and altered architecture of the sclera leads to increased axial elongation [4]. Scleral remodeling results from alterations in the composition of collagen content, which is regulated by both matrix metalloproteinases (MMP) and tissue inhibitors of metalloproteinases (TIMP) [5-10]. Hepatocyte growth factor (*HGF*) and its receptor, *C-MET*, modulate MMP and TIMP pathways and therefore may play an active role in scleral remodeling, axial elongation, and myopia development [11-13].

The contribution of genetic factors to the development of myopia has been firmly established by previous studies and has been extensively summarized in review publications [14,15]. *HGF* and *C-MET* are two myopia candidate genes studied in Asian populations ([16]; personal communication, Khor C.C., Agency for Science, Technology, and Research, Singapore; December, 2007). Han et al. [16] performed family-based association analysis of *HGF* polymorphisms (3 tagging SNPs) in a study cohort of 128 Han Chinese nuclear families with highly myopic siblings that found association between rs3735520 (*HGF*5b-b) and high myopia. However, Wang et al. [17] were not able to replicate this association in a recent case-control (288 cases and 208 controls) study using the same ethnic population. In unpublished data from Khor C.C., a case-control study found the *C-MET* SNP rs2073560 (MET+110703) was associated with moderate myopia in 650 Singaporean school children. The study genotyped and analyzed 12 *C-MET* SNPs.

To date, the role of *HGF* and *C-MET* in association with refractive error has not been investigated in Caucasians. Myopia is a complex, multi-factorial disorder. The fact that multiple genes and environmental factors influence myopia development necessitates determination of potential candidate gene involvement in independent and/or diverse datasets. Therefore, we ascertained and genotyped Caucasian family subjects biased towards high myopia and performed association analyses for polymorphisms and haplotypes of *HGF* and *C-MET* with multiple myopic refractive error phenotypes.

## Methods

### Subject selection

Approval was obtained from the Institutional Review Boards at both participating institutions, the Duke University Medical Center (Durham, NC) and the Children’s Hospital of Philadelphia (Philadelphia, PA). Informed consent was acquired from all subjects prior to entering the study and the principles of the Declaration of Helsinki were followed. Families were eligible for inclusion in the study if they had one member with spherical refractive error of -5.00 D or more. All available family members were recruited. Patients with systemic conditions, syndromic disorders, or ophthalmologic conditions which could predispose to high myopia were excluded. Subjects underwent a complete ophthalmic examination including cycloplegic retinoscopy and/or autorefraction (Topcon RM-8800; Topcon Medical Systems Inc., Paramus, NJ).

### Marker selection and genotyping

**Figure 1:**
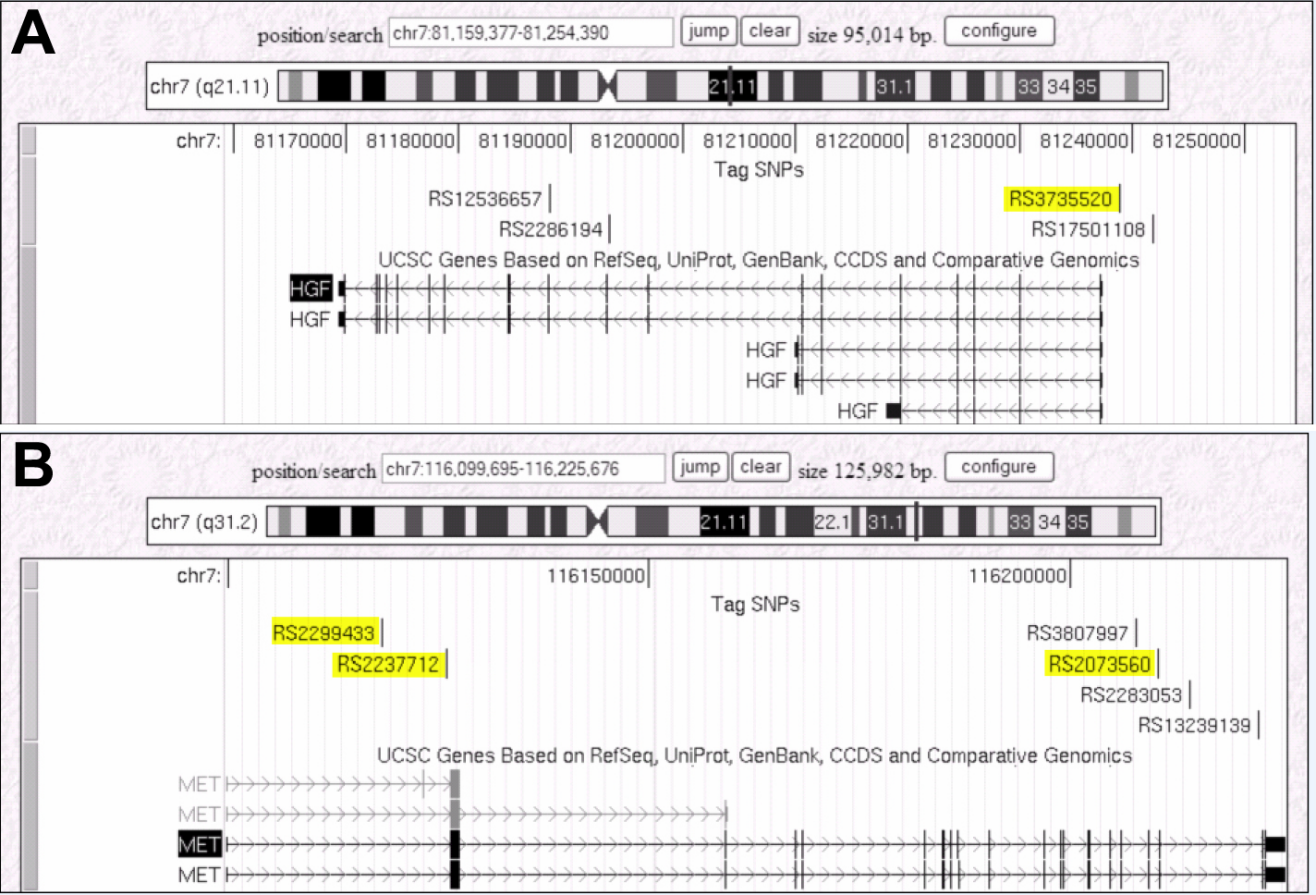
Schematic representations of the SNPs selected for the *HGF* (1A) and *C-MET* (1B) genes (source UCSC genome browser). A total of 4 SNPs were selected for *HGF* and 6 were selected for *C-MET*. The SNPs highlighted in yellow have previously been associated with high myopia as reported by Han et al. [16] for *HGF* and Khor et al. [Personal communication, Khor CC] for *C-MET*. All of the SNPs are intronic with the exception of rs3735520 which is located 1.5KB upstream from *HGF* and rs17501108 which is located 4.5KB upstream from *HGF.*

For the candidate genes, *HGF* and *C-MET,* the SNPSelector® program [18] was used to choose tagging SNPs that met the criteria of a Pearson squared correlation (r^2^) threshold of 0.76 in the linkage disequilibrium bins and a minor allele frequency (MAF) greater than 5% in the Caucasian population. The SNPs that Han et al. [16] and Khor C.C. (Personal communication) reported to have significant association with myopia were included. Sufficient tagging SNPs were selected to provide full gene coverage. The SNPs chosen for the candidate genes are shown in Figure 1.

Following venous blood sample collection, genomic DNA was extracted using AutoPure LS^®^ DNA Extractor and PUREGENE™ reagents (Gentra Systems Inc; Minneapolis, MN). For genotyping reactions, custom TaqMan^®^ allelic discrimination assays, consisting of unlabeled polymerase chain reaction (PCR) primers and the TaqMan^®^ minor groove binding group (MGB) probes (FAM™ and VIC^®^ dye-labeled; Applied Biosystems, Foster City, CA) were utilized. The assays were comprised of 2 unlabeled PCR primers and 2 allele-specific probes. PCR reactions were performed with Taqman^®^ Universal PCR Master Mix on the GeneAmp^®^ PCR System 9700 (Applied Biosystems). The ABI7900HT Fast PCR System (Applied Biosystems) was employed to read the allelic discrimination calls.

For quality control (QC) purposes, two Centre d’Etude du Polymorphisme Humain (CEPH) DNA standards were included in each 96 well plate, and two blind duplicate samples were used per plate. Genotype submission to the analysis database required 100% matching QC genotypes within and across plates and at least 95% genotyping efficiency. All genotypes were in Hardy-Weinberg equilibrium. Mendelian inconsistencies were checked and resolved.

### Statistical analyses

Analyses compared the following myopic refractive error phenotypes: 1) high myopia (≤-5.00 D), 2) mild to moderate myopia (-0.50 to -5.00 D), 3) any myopia (≤-0.50 D), and 4) extreme high myopia (as defined in Han et al. [16]; ≤-10.00 D) versus emmetropia. Phenotypes were based either on sphere (SPH) or spherical equivalent (SE=sphere + [cylinder/2]). The family-based pedigree disequilibrium test (PDT) [19], genotypic PDT (genoPDT) [20], and the association test in the presence of linkage (APL) were performed for each myopic phenotype state [21]. The number of affected and unaffected subjects for each classification is described in detail in Table 1. Table 2 provides clinical information of the different myopic phenotypes.

**Table 1:** Summary of myopia definitions and the number of subjects in each group for the study population.

**Phenotype**	**Definition based on myopia severity**	**Type of refractive error**	**Affected (A)**	**Unaffected (N)**	**Unknown (U)**	**Total**
**High-grade myopia**	A<-5.0 D N≥-0.49 D U=others	SPH	303	137	209	649
		SE	285	155	209	649
**Mild to moderate myopia**	-4.99 D≤A≤-0.5 D N≥-0.49 D U=others	SPH	206	137	306	649
		SE	204	155	290	649
**Any myopia**	A<-0.5 D N>0 D -0.5 D<U<0 D	SPH	509	124	16	649
		SE	489	131	29	649
**Extreme myopia**	A≤-10.0 D N≥-0.5 D -10.0 D<U<-0.5 D	SPH	128	137	384	649
		SE	113	155	381	649

A=affected, N=unaffected, U=unknown, D=diopters, SPH=sphere, SE=spherical equivalent.

**Table 2:** Summary of the refractive error data for phenotypes as defined by sphere.

		High myopia			Mild-moderate myopia			Any myopia		
		Affected	Not affected	Unknown	Affected	Not affected	Unknown	Affected	Not affected	Unknown
Number (n)		303	137	209	206	137	306	509	124	16
Age, years (mean±SD)		36.3±17.4	40.4±23.0	40.1±20.9	40.1±20.9	40.4±23.0	36.3±17.5	37.8±18.9	41.8±23.4	30.5±17.6
Male (n, %)		148 (49%)	66 (48%)	103 (49%)	101 (49%)	66 (48%)	150 (49%)	249 (49%)	62 (50%)	6 (38%)
Sphere, diopters (mean±SD)	OD	-9.75±5.16	0.77±1.44	-2.14±1.41	-2.15±1.41	0.77±1.44	-9.7±5.19	-6.67±5.53	0.86±1.48	-0.3±0.44
	OS	-9.73±4.85	0.78±1.71	-2.09±1.31	-2.11±1.31	0.78±1.71	-9.67±4.89	-6.64±5.36	0.78±1.44	0.58±3.24
Spherical equivalent, diopters (mean±SD)	OD	-9.19±5.11	0.98±1.83	-1.77±1.38	-1.77±1.38	0.98±1.83	-9.19±5.11	-6.16±5.44	0.98±1.55	0.96±3.66
	OS	-9.2±4.84	0.81±1.46	-1.75±1.29	-1.75±1.29	0.81±1.46	-9.2±4.84	-6.17±5.29	0.89±1.51	-0.02±0.21

Statistical significance for PDT, genotypic PDT and APL tests was defined as a p-value ≤0.05 with subsequent Bonferroni correction for multiple testing (=0.05/number of markers tested), 0.0125 for *HGF* and 0.008 for *C-MET.* Two and three-marker sliding window for haplotype association tests were performed using APL. For the global test statistic generated by haplotype association testing, a p-value of ≤0.01 (0.05/number of haplotype windows) was considered statistically significant. Statistical significance for individual haplotype association testing was defined as a p-value ≤0.007 for three loci (0.05/maximum number of haplotypes for each group of loci) and ≤0.0125 for two loci.

## Results

The *HGF* results are categorized by myopic refractive error definition in the following section. Table 3 summarizes the APL and PDT analyses for *HGF* SNPs. Table 4 depicts the APL and PDT analyses for the *C-MET* SNPs.

**Table 3:** APL and PDT analyses of the *HGF* gene tagging polymorphisms.

**Marker**	**High myopia**				**Mild-moderate myopia**				**Any myopia**				**Very high myopia**			
**SPH**		**SE**		**SPH**		**SE**		**SPH**		**SE**		**SPH**		**SE**	
**APL**	**PDT**	**APL**	**PDT**	**APL**	**PDT**	**APL**	**PDT**	**APL**	**PDT**	**APL**	**PDT**	**APL**	**PDT**	**APL**	**PDT**
rs12536657	0.1346	1.0000	0.1287	0.6002	0.0431	0.0637	0.0432	0.0776	0.1151	0.1853	0.0995	0.2053	0.1363	0.2858	0.1253	0.6121
rs17501108	0.5487	0.5930	0.4154	0.2482	0.0319	0.2087	0.0510	0.3980	0.0704	0.1060	0.0794	0.1701	0.4512	0.2568	0.2250	0.1025
rs2286194	0.0396	0.9081	0.0613	1.0000	0.5502	0.5637	0.3730	0.4328	0.2997	0.7180	0.2037	0.7995	**0.0058**	0.7963	**0.0041**	0.7815
rs3735520	0.2390	0.8514	0.1888	0.9223	**0.0028**	0.0874	**0.0056**	0.1486	0.0171	0.2964	0.0131	0.3304	0.1734	0.4795	0.0343	0.2971

Analyses were based on the following phenotypes using either sphere (SPH) or spherical equivalent (SE): 1) high myopia (≤-5.00 diopters [D]) 2) mild to moderate myopia (-0.50 to -5.00 D), 3) any myopia (≤-0.50 D) and 4) very high myopia (≤-10.00 D) versus emmetropia. A p-value of ≤0.0125 was considered statistically significant (in bold). Alternatively, corrected p-values for 0.0028, 0.0056, 0.0058 and 0.0041 were 0.0112, 0.0224, 0.0232, and 0.0164, respectively.

**Table 4:** APL and PDT analyses of the *C-MET* gene tagging polymorphisms.

**Marker**	**High myopia**				**Mild-moderate myopia**				**Any myopia**				**Very high myopia**			
**SPH**		**SE**		**SPH**		**SE**		**SPH**		**SE**		**SPH**		**SE**	
**APL**	**PDT**	**APL**	**PDT**	**APL**	**PDT**	**APL**	**PDT**	**APL**	**PDT**	**APL**	**PDT**	**APL**	**PDT**	**APL**	**PDT**
rs13239139	0.2750	0.6506	0.2660	0.6506	0.5012	0.7009	0.4469	1.0000	0.2011	0.9230	0.2290	0.8488	0.9552	0.0614	0.7363	0.1390
rs2073560	0.1595	0.6976	0.1750	0.7653	0.5270	0.8084	0.3578	0.9013	0.0943	0.5839	0.1387	0.5870	0.7220	0.2636	0.4383	0.5221
rs2237712	0.3065	0.4054	0.3283	0.4054	0.0868	0.2733	0.1087	0.3359	0.6348	0.1599	0.6616	0.1944	0.0658	0.1573	0.0599	0.1573
rs2283053	0.2117	0.9136	0.2175	1.0000	0.3869	0.7180	0.2905	0.8055	0.2186	0.6892	0.2552	0.6921	0.6237	0.0522	0.8450	0.1172
rs2299433	0.9420	0.3994	0.9562	0.3173	0.1589	0.2965	0.0952	0.3074	0.1983	0.7335	0.2146	0.7932	0.7538	0.1495	0.8000	0.1573
rs3807997	0.2103	1.0000	0.1979	0.9136	0.5249	0.8084	0.3423	0.9013	0.1240	0.8445	0.1916	0.8460	0.8910	0.0614	0.8350	0.1390

Analyses were based on the following phenotypes using either sphere (SPH) or spherical equivalent (SE): 1) high myopia (≤-5.00 diopters [D]) 2) mild to moderate myopia (-0.50 to -5.00 D), 3) any myopia (≤-0.50 D) and 4) very high myopia (≤10.00 D) versus emmetropia.

### Mild to moderate myopia

On APL analysis, the risk allele T for the *HGF* SNP, rs3735520, was found to have a statistically significant association with mild to moderate myopia versus emmetropia (p=0.002768 for SPH and 0.005609 for SE). For this SNP, the p-values with PDT were not statistically significant (p=0.0874 for SPH and 0.1486 for SE). Two other *HGF* SNPs, rs12536657 and rs17501108, also yielded promising associations with APL testing (p=0.043 for both SPH and SE and p=0.032 for SPH and 0.051 for SE). The PDT for rs12536657 (p=0.0637 for SPH and 0.0776 for SE) and rs17501108 (p=0.2087 for SPH and 0.398 for SE) did not reveal any statistically significant associations.

The global test statistic for sliding window APL analysis based on two and three marker loci yielded two significant haplotypes: rs12536657-rs2286194 (p=0.0042 for SPH and 0.0055 for SE) and rs2286194-rs3735520-rs17501108 (p=0.0074 for SPH and 0.0627 for SE). Table 5 shows the two and three marker haplotype association results for mild to moderate myopia. Under the significant finding with global haplotype testing, the specific haplotype of G-A for rs12536657-rs2286194 (p=0.001499 for SPH and 0.001826 for SE) and A-C-G for rs2286194-rs3735520-rs17501108 (p=0.006571 for SPH) were determined (Table 6). The haplotype G-A for rs1253667-rs2286194 showed the strongest association for mild to moderate myopia.

**Table 5:** Association analysis between haplotypes phenotypes.

**Markers**	**High myopia**		**Mild-moderate myopia**		**Any myopia**		**Extreme myopia**	
	**SPH**	**SE**	**SPH**	**SE**	**SPH**	**SE**	**SPH**	**SE**
rs12536657 rs2286194	0.6369	0.5721	**0.0042 (0.021)**	**0.0055 (0.0275)**	**0.0094 (0.047)**	0.0152	0.4933	0.3288
rs2286194 rs3735520	0.2958	0.2810	0.0342	0.1044	0.0358	0.0437	0.0871	0.0733
rs3735520 rs17501108	0.0800	0.0766	0.0289	0.0410	**0.0062 (0.031)**	**0.0075 (0.0375)**	0.1447	0.0481
rs12536657 rs2286194rs3735520	0.2871	0.2844	0.0751	0.1107	0.0384	0.0345	0.3075	0.1607
rs2286194 rs3735520rs17501108	0.1634	0.1577	**0.0074 (0.037)**	0.0627	**0.0092 (0.046)**	**0.0057 (0.0285)**	0.1661	0.0571

Association analysis between haplotypes and the following phenotypes using either sphere (SPH) or spherical equivalent (SE): 1) high myopia (≤-5.00 diopters [D]) 2) mild to moderate myopia (-0.50 to -5.00 D), 3) any myopia (≤-0.50 D) and 4) very high myopia (≤10.00 D) versus emmetropia. Significant global haplotypes were obtained by analyzing the possible allele combinations of either two or three loci. Locus nucleotide identity and p-value are identified for each combination with a significant p-value. Corrected p-values are in parentheses.

**Table 6:** Allele frequencies for the haplotypes found to be significant under global testing for mild-moderate myopia (-0.50 to -5.00D).

**Haplotype**			**Frequency**	**Observed**	**Expected**	**p value**	**Corrected p value**
rs12536657	rs2286194						
A	A		0.1557	14	19	0.0758	0.3032
A	T		0.0059	1	1	0.3025	1.21
G	A		0.7309	111	101	**0.0015**	**0.006**
G	T		0.1075	8	13	0.0215	0.86
rs2286194	rs3735520	rs17501108					
A	C	G	0.3142	49	43	**0.0066**	**0.0462**
A	C	T	0.3952	42	48	0.0734	0.5138
A	T	G	0.1538	22	18	0.0753	0.5271
A	T	T	0.0138	2	2	0.4538	3.1766
T	C	T	0.1230	9	14	0.0251	0.1757

Significant p values are in bold.

### High myopia

APL testing for the *HGF* SNP, rs2286194, yielded a p-value that did not meet a significant level (p=0.0396 for SPH and 0.0613 for SE) when comparing the high myopia with emmetropia groups. For this marker, the PDT p-values were not significant (p=0.8815 for SPH and 1 for SE). Sliding window APL analysis with the *HGF* markers also did not reveal any significant haplotype associations.

### Extreme high myopia

The *HGF* SNP, rs2286194, was found to have a statistically significant association with extreme high myopia when compared with emmetropia by APL testing (p=0.0058 for SPH and 0.0041 for SE). The risk allele for rs2286194 was A. The PDT p-values for this SNP were not significant (0.7963 for SPH and 0.7815 for SE). There was no association identified for rs3735520 by APL analysis (0.1734 for SPH and 0.03433 for SE) or PDT analysis (p=0.4795 for SPH and 0.2971 for SE). None of the other *HGF* SNPs were observed to have statistically significant association with extreme high myopia compared to emmetropia by APL or PDT analyses. Sliding window APL analysis with *HGF* markers did not demonstrate any significant haplotypes for this phenotype.

### Any myopia

Promising association was found between any myopia and rs3735520 (p=0.01714 for SPH and 0.01308 for SE). However, no individual SNPs were found to be statistically significant under the most conservative definition of significance.

The global test statistic for sliding window APL analysis based on two and three marker loci found 3 significant haplotypes: rs12536657-rs2286194 (p= 0.009404 for SPH and 0.01519 for SE), rs3735520-rs17501108 (p=0.006182 for SPH and 0.00752 for SE), and rs2286194-rs3735520-rs17501108 (p=0.009198 for SPH and 0.005702 for SE). Similar to mild to moderate myopia, the haplotype G-A for rs12536657-rs2286194 showed the strongest association for any myopia (p=0.003291 for SPH and 0.004621 for SE). Table 7 summarizes the statistically significant findings for the mild and moderate and the any myopia groups.

**Table 7:** Summary of the significant individual markers, global haplotypes and specific haplotypes for mild to moderate myopia (-0.50 to -5.00 D) and any myopia (≤-0.50 D).

		**Mild-moderate myopia**		**Any myopia**	
		**SPH**	**SE**	**SPH**	**SE**
**Individual Markers**					
rs12536657		0.0431	0.0432	0.1151	0.0995
rs17501108		0.0319	0.0510	0.0704	0.0794
rs2286194		0.5502	0.3730	0.2997	0.2037
rs3735520		**0.0028 (0.0112)**	**0.0056 (0.0224)**	0.0171	0.0131
**Global Haplotypes**					
rs12536657 rs2286194		**0.0042 (0.021)**	**0.0055 (0.0275)**	**0.0094 (0.047)**	0.0152
rs3735520 rs17501108		0.0289	0.0410	**0.0062 (0.031)**	**0.0075 (0.0375)**
rs2286194 rs3735520rs17501108		**0.0074 (0.037)**	0.0627	**0.0092 (0.046)**	**0.0057 (0.0285)**
**Specific Haplotypes**					
rs12536657 rs2286194	G-A	**0.0015 (0.006)**	**0.0018 (0.0072)**	**0.0033 (0.0132)**	**0.0046 (0.0184)**
rs2286194 rs3735520rs17501108	A-C-G	**0.0066 (0.0462)**	0.0191	0.0231	0.0198

Corrected p values are in parentheses.

## Discussion

The present study examined the associative involvement of the *HGF* and *C-MET* with mild to moderate, high, and extreme high myopia phenotypes using a large Caucasian family-based cohort biased toward high myopia. We did not find associations between myopia and *C-MET* polymorphisms, and hence, the gene does not appear to play a role in myopia pathogenesis in our dataset. *HGF* demonstrated the most promising findings with the following evidence to support its association: (1) the significant p-values met the family-wide significance level based on Bonferroni correction (minimum p=0.0028 at rs3735520); (2) the association signal was consistent between mild to moderate and any myopia; (3) regardless of phenotype definition, SNPs in *HGF* showed some indication of association, even though it was not always from the same markers; and (4) the results were similar with the different phenotype definitions, SE and SP. In addition to these significant analytical results, *HGF* is an excellent biological candidate gene for myopia as it potentially plays a role in the scleral remodeling process.

The mild to moderate myopia group showed a statistically significant association with the *HGF* SNP, rs3735520. On global testing, this SNP was included in the haplotype, rs22861947-rs3735520-rs17501108, which had a statistically significant association with mild to moderate myopia as well. The specific haplotype, A-C-G, demonstrated a significant association for this phenotype. These findings indicate that rs3735520 may be an important SNP in the development of mild to moderate myopia.

In addition, two other *HGF* SNPs, rs12536657 and rs17501108, had promising associations with this phenotype. By conducting haplotype analysis for all combinations of two and three loci, we found that the combination including rs12536657-rs2286194 revealed the most significant haplotype association. Although rs12536657 and rs2286194 only met a nominal significance (p≤0.05), they appeared to act together to generate a stronger association than the single markers alone. Therefore, in addition to rs3735520, the region surrounding rs12536657 and rs2286194 may harbor additional important variants for this phenotype.

The association of *HGF* with myopia was first described by Han et al., who defined high myopia with SE >-10.00 D. In their study, significant association was found between rs3735520 and high-grade myopia. However, we have identified a different SNP association, rs2286194, which is 45,490 base pairs upstream of rs3735520, for both high and extreme high myopia. In our study, rs3735520 had a “marginal” association with extreme high myopia, but not high myopia.

The discrepancy between the results of the two studies may have occurred because our study focused strictly on the Caucasian population, whereas Han et al. [16] performed his study in a Han Chinese population. In addition, the two studies used different grades of myopia as inclusion criteria. Our inclusion criteria was myopia ≤-5.00 D which resulted in a smaller extreme high myopia sample size, while Han et al. [16] targeted subjects with myopia ≤-10.00 D. Another possible consideration is that patients in our study were ascertained earlier in the process of developing myopia, and may proceed to higher grades of myopia with time. Since Han et al. [16] did not stratify their results by level of myopia as we did, we cannot verify the association evidence for rs3735520 in mild to moderate myopia. Due to significant differences in study design, it is not surprising that meta-analyses for rs3735520 and rs2286194 using Fisher’s exact test were not significant (0.0627 and 0.0863, respectively).

It should be noted that the evidence of significant association presented here is from the APL analysis, and PDT did not reveal any significant results. While both PDT and APL are robust family-based association methods, it is known that PDT treats each discordant sibpair and parent-offspring triad independently within a general pedigree, while APL considers identical by descent from pairs of siblings. Therefore, APL is generally more robust than PDT when multiple affected individuals per family are available. Since our ascertainment strategy was initially targeted on linkage study, the majority of families used in the present study have more than two affected individuals and 8% families have more than three generations, which is a more suitable study design for APL than PDT tests. This may be the reason that there was a discrepancy between the results from the two methods.

In conclusion, our study revealed an association between rs3735520 and 2 haplotypes and mild-moderate myopia in a predominately Caucasian population. We also detected an association between rs2286194 and extreme high myopia. We did not find any significant associations between *C-MET* polymorphisms and myopia. This represents the first analysis of *HGF* and *C-MET* polymorphisms and myopia in a non-Asian cohort. Our study suggests that the *HGF* associations with myopia may be present in the Caucasian population, but that genotype-phenotype relationships may differ between various ethnic datasets in the complex disorder of myopia. Further genetic studies in Caucasians are needed to confirm our findings.
